# Combined Long-Period Fiber Grating and Microcavity In-Line Mach–Zehnder Interferometer for Refractive Index Measurements with Limited Cross-Sensitivity †

**DOI:** 10.3390/s20082431

**Published:** 2020-04-24

**Authors:** Monika Janik, Marcin Koba, Krystian Król, Predrag Mikulic, Wojtek J. Bock, Mateusz Śmietana

**Affiliations:** 1 Institute of Microelectronics and Optoelectronics, Warsaw University of Technology, Koszykowa 75, 00-662 Warszawa, Poland; mkoba@elka.pw.edu.pl (M.K.); K.Krol@imio.pw.edu.pl (K.K.); M.Smietana@elka.pw.edu.pl (M.Ś.); 2 Department of Metrology and Optoelectronics, Faculty of Electronics, Telecommunications and Informatics, Gdansk University of Technology, Narutowicza 11/12, 80-233 Gdansk, Poland; 3 The National Institute of Telecommunications, Szachowa 1, 04-894 Warszawa, Poland; 4 Centre de Recherche en Photonique, Université du Québec en Outaouais, 101 Rue St Jean Bosco Gatineau, Gatineau, QC J8X 3X7, Canada; predrag.mikulic@uqo.ca (P.M.); wojtek.bock@uqo.ca (W.J.B.)

**Keywords:** Mach–Zehnder interferometer (MZI), long-period grating (LPG), optical fiber sensors, refractive index sensing, femtosecond laser micromachining, thin films, multiparameter sensing, cross-sensitivity

## Abstract

This work discusses sensing properties of a long-period grating (LPG) and microcavity in-line Mach–Zehnder interferometer (µIMZI) when both are induced in the same single-mode optical fiber. LPGs were either etched or nanocoated with aluminum oxide (Al_2_O_3_) to increase its refractive index (RI) sensitivity up to ≈2000 and 9000 nm/RIU, respectively. The µIMZI was machined using a femtosecond laser as a cylindrical cavity (d = 60 μm) in the center of the LPG. In transmission measurements for various RI in the cavity and around the LPG we observed two effects coming from the two independently working sensors. This dual operation had no significant impact on either of the devices in terms of their functional properties, especially in a lower RI range. Moreover, due to the properties of combined sensors two major effects can be distinguished—sensitivity to the RI of the volume and sensitivity to the RI at the surface. Considering also the negligible temperature sensitivity of the µIMZI, it makes the combination of LPG and µIMZI sensors a promising approach to limit cross-sensitivity or tackle simultaneous measurements of multiple effects with high efficiency and reliability.

## 1. Introduction

For many chemical and biological applications, precise monitoring of the refractive index (RI) of a liquid analyte is of great importance [1]. However, the highly accurate RI measurements depend on many factors such as temperature (T) cross-sensitivity, especially for on-site applications, or surface sensitivity during the detection of small molecules as DNA targets, which can be easily disturbed by the T and the RI fluctuations, giving false results. In recent years, various fiber optical platforms incorporating multiple sensor elements have been used for multi-parameter sensing, especially for simultaneous measurements of the RI and the T. These include a reflection-mode long-period grating (LPG) with an intrinsic Fabry–Perot interferometer (FPI) [2], an FPI integrated with a fiber Bragg grating (FBG) [3], a dual LPG [4], a core-offset Mach–Zehnder interferometer (MZI) combined with FBG [5], or an FBG with microcavity in-line MZI (µIMZI) [6]. A review on this topic was presented by S. Pevec and D. Donlagic [7]. Each of these sensors has its limitations, e.g., the FBG-µIMZI offers very high RI sensitivity, compact size, and precise sensing element location, but during simultaneous RI and T measurements, the wavelength shift obtained for the interference minimum of the µIMZI is approximately two orders of magnitude greater than the one for the resonant wavelength of the FBG. Thus, simultaneous tracing of the two shifts corresponding to RI and T was difficult and substantially limits practical applications of this platform. To overcome such issues, in this work we propose a sensor based on two the most RI sensitive optical fiber devices, namely the LPG and the µIMZI. Both sensing concepts have been known and explored for decades for a great variety of sensing applications [8,9]. 

The LPG is a periodic modulation of the RI along the length of the core in a single-mode optical fiber, which allows for coupling between the fundamental core mode and a series of cladding modes [10,11]. The coupling results in the appearance of resonances in the LPG transmission spectrum, where each of the resonances corresponds to the coupling of a certain cladding mode. The sensitivity of the LPG is typically defined as a spectral shift of the resonance wavelength with a measurand. Moreover, enhancing the LPG’s RI sensitivity has been also explored and reported. It has been improved through tuning its working point [12] and coating the grating with a high-RI nano-overlay [13]. Thus, currently, LPGs offer sensitivity exceeding 10,000 nm/RIU for measurements in proximity to the sensor’s surface [14]. However, because of its high-temperature sensitivity, there is always a measurement error resulting from the cross-sensitivity [15]. To counter this problem, a control over T of the investigated liquid during the measurements is typically applied [16]. 

According to the MZI concept, the light beam splits into two parts propagating along two different paths, which interfere with one another after a certain distance [17]. When guiding conditions in one of the paths are disturbed, the effect can be identified by tracking changes in the interference pattern. The µIMZI structures presented in this paper are fabricated in a standard telecom optical fiber using femtosecond laser ablation [17], where the light propagates in the fiber core and splits at the cavity wall into reference (core) and sensing (cavity) beams, each with different optical lengths. When the beams reach the opposite wall of the cavity, they interfere like the two beams in an MZI. Thanks to the size reaching tens of micrometers and application of fs laser micromachining, the device is compact, highly reproducible, and well-suited for applications where as low as pL volumes are investigated. Moreover, it may offer negligible T sensitivity and very high RI sensitivity reaching over 20,000 nm/RIU [17,18].

In this paper, we discuss an ability for combining the two sensing devices, namely LPG and µIMZI, into a single compact sensing system. Based on the sensing scheme proposed here, multiple measurands can be calculated from a spectrum acquired using a single spectrum analyzer. The two optical fiber sensors are both capable of high-sensitivity RI measurements with one major difference: the LPG is best suited for RI measurements close to the fiber’s surface while the µIMZI has been applied in bulk RI measurements, where the surface effect can be ignored [19]. When, e.g., biological molecules from a liquid sample under analysis attach to the surface of a functionalized sensor, the observed output signal corresponds to the growth of a biological layer. This sensing phenomenon and the effect of surface RI sensitivity often form the basis for label-free sensing using LPGs [20,21]. Although it is proven that for LPG both types of RI sensitivities can be optimized to some extent, it applies only for simple RI measurements using liquids. Since there is no difference between liquid properties at the surface and away from the LPG, the same measurement results are correct for RI in volume. However, when only thin biofilm at the LPG’s surface needs to be identified the measurements gain complexity. In this case, the result of the measurement is a superposition of RI changes at the surface and in the volume, and at this point, only these at the surface are of interest. That is why for this exact application, we propose to discriminate these two kinds of sensitivity using LPG response associated mainly with the surface RI sensitivity (S_S_), and the µIMZI response with the volume RI sensitivity (S_v_). At washing steps, only the response from LPG will change due to the appearance of thin biofilm, while part of the spectrum corresponding to µIMZI will stay unchanged. Thus, the sensing configuration allows for clear identification of the changes at the surface. Considering also the negligible T sensitivity of the µIMZI, the combination of LPG and µIMZI sensors makes a promising approach to tackle simultaneous measurements of multiple effects with high efficiency and reliability. 

## 2. Materials and Methods

### 2.1. Fabrication of LPG 

**Figure 1:**
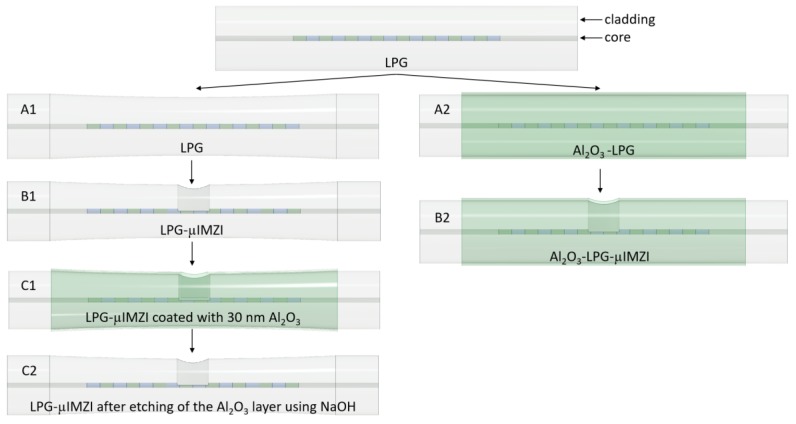
Schematic visualization of the sample preparation, where (**A1**) represents a long-period grating (LPG) etched with hydrofluoric (HF) acid; (**A2**)—an LPG nanocoated with aluminum oxide (Al_2_O_3_); (**B1**)—a combination of the microcavity in-line Mach–Zehnder interferometer (µIMZI) and etched LPG; (**B2**)—a combination of the µIMZI and nanocoated LPG; (**C1**)—a LPG-µIMZI coated with 30 nm Al_2_O_3_ layer as a simulation of a biofilm formation; (**C2**)—a LPG-µIMZI after etching of the Al_2_O_3_ layer with sodium hydroxide. Features are not to scale.

A set of LPGs was fabricated in hydrogen-loaded germanium-doped Corning SMF-28 single-mode optical fibers by UV irradiation of a 5-cm long fiber section with a KrF excimer laser [22]. The fibers were exposed through a chromium amplitude mask with a period of 226.8 μm. The period of the grating has been optimized to reach the RI sensitivity around 2000 nm/RIU within the RI range between 1.333 and 1.350 RIU, and to obtain dispersion turning point (DTP) between 1500 and 1600 nm [23]. After the UV-writing, the LPGs were annealed at 150 °C for 3 h to release the hydrogen and to stabilize the properties of the gratings. Next, a set of LPGs was chemically etched in hydrofluoric (HF) acid up to the DTP taking place at an external RI = 1.3330 RIU (Figure 1A1) [20]. Considering the etching rate of 10% HF acid [2] and the etching time, it is estimated that changes in the cladding thickness can reach up to 5 µm. Thus, the final diameter of the cladding decreased to ca. 115 µm. The estimated values have been confirmed by simulations [20,24]. The other set of LPGs was coated with aluminum oxide (Al_2_O_3_) nano-overlay according to the procedure reported in [14] (Figure 1A2). This nanocoating with a high-RI material allowed us to optimize RI sensitivity by obtaining both mode transition (MT) and DTP effects [12].

### 2.2. Fabrication of μIMZI

The cylindrical cavity (60 µm diameter) was micromachined in the middle of the LPG through the cladding and core reaching half of the fiber (60 µm depth) (Figure 1B1,B2). The structure has a circular cross-section, flat bottom, while its walls, with good approximation, are perpendicular to the fiber’s long axis [25]. The cavity diameter of 60 μm was chosen due to mechanical constraints and for ease of liquid application. Micromachining was performed using a Solstice Ti:Sapphire fs laser operating at λ = 795 nm. The fiber was irradiated by 82 fs pulses. To make the microcavity, the laser beam was directed into a suitably designed micromachining setup based on the Newport μFab system. The system was equipped with a 20× lens, with NA = 0.30. During the fabrication, the fiber’s transmission spectrum was monitored with an NKT Photonics SuperK COMPACT supercontinuum white light source and a Yokogawa AQ6370C optical spectrum analyzer. The fabrication process was controlled with software developed in-house. 

### 2.3. LPG and LPG-μIMZI Analysis

**Figure 2:**
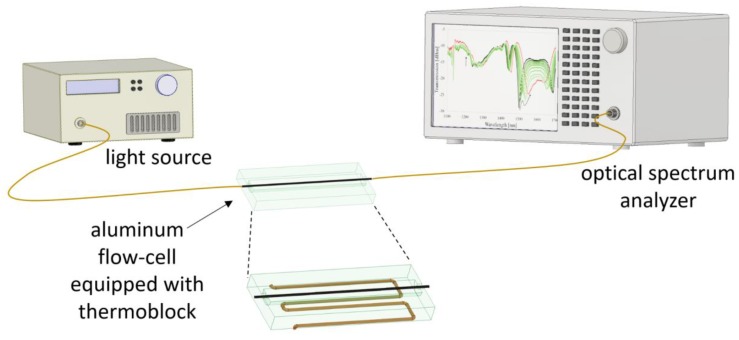
Schematic representation of the measurement setup.

First, the spectral response of the LPG was investigated in the wavelength range from 1100 to 1700 nm using the light source and spectrum analyzer described in Section 2.2. The RI sensitivity was identified by immersing the LPGs in water/glycerin mixture with RI ranging from 1.3330 to 1.4000 RIU. The RIs of the liquids were measured using a VEE GEE PDX-95 digital refractometer with a resolution of 10^−4^ RIU. Before the first immersion in the liquids, the Al_2_O_3_-nanocoated LPG-μIMZI underwent 30 s long oxygen plasma processing, which increased the wettability of the film’s surface and made filling the cavity with the liquids possible [17]. The RI of liquids was verified just after the transmission measurements by taking them directly from the vicinity of the fiber sample. Between immersions, the LPG was rinsed with deionized water. T and strain were kept constant during all the RI measurements. The T measurements were performed while the structures were immersed in water. We used a home-made aluminum flow-cell where the bottom part was T-controlled in the range 10–45 °C. A reference μIMZI structure also underwent the same testing procedure. Figure 2 depicts the schematic representation of the measurement setup. The measurements were conducted on three etched and three nanocoated LPGs with fabricated microcavities. Each measurement was conducted in five repetitions to check the stability of the sensor and measurement setup. The following results present the representative spectra averaged from five consecutive measurements of two chosen sensors, namely LPG-µIMZI and Al_2_O_3_-LPG-µIMZI.

### 2.4. Monitoring of RI Changes at the Surface with LPG-µMZI.

The 30 nm in thickness Al_2_O_3_ nano-overlay was deposited on LPG-µMZI following the procedure described in [10] (Figure 1C1). Next, the Al_2_O_3_ thickness was reduced on LPG and the microcavity by the sensor immersion in a sodium hydroxide (NaOH) of known concentration (10 mM and 1 M) at a fixed temperature (T = 20 °C) for a specified time [26], followed by extensive washing with deionized water (Figure 1C2). The optical transmission spectrum of the µIMZI was investigated during the etching and compared when the sensor was immersed in deionized water. Before the first NaOH etching, the cavity underwent 30-s long oxygen plasma processing, as mentioned in Section 2.1.

## 3. Results and Discussion

### 3.1. RI Sensing with LPG or µMZI Structure

**Figure 3:**
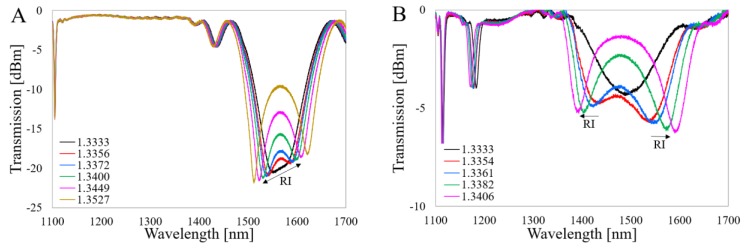
Spectral response of the investigated structures (**A**) LPG and (**B**) Al_2_O_3_-LPG to changes in refractive index (RI). The arrows indicate a spectral shift with external RI.

The spectral responses of the LPG and Al_2_O_3_-nanocoated LPG (Al_2_O_3_-LPG) to RI before μIMZI micromachining are shown in Figure 3A,B, respectively. An increase of the spectral distance between the resonances is observed when the RI increases, which is very characteristic for this type of sensing structure working at the DTP of higher-order cladding modes [14]. The RI sensitivity for each resonance is close to 2000 and 9000 nm/RIU for LPG and Al_2_O_3_-LPG, respectively. Due to the higher RI sensitivity of the Al_2_O_3_-LPG, the resonances shift significantly more with RI.

**Figure 4:**
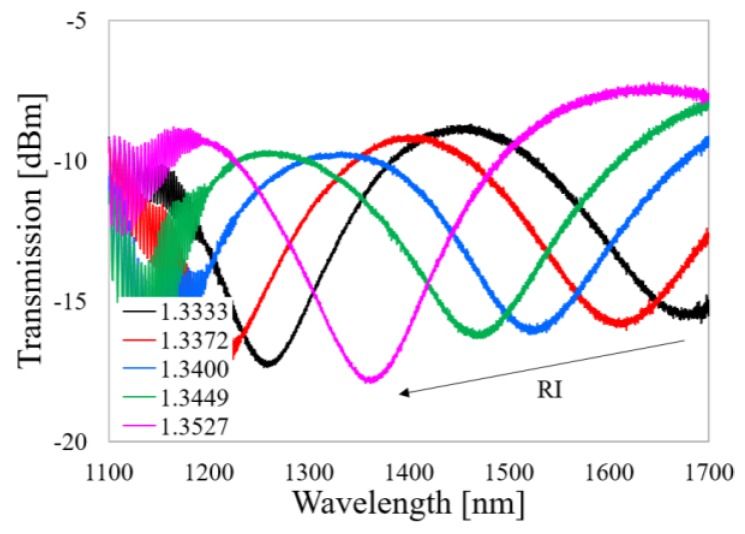
Spectral response of the reference µIMZI with cavity diameter *d* = 60 µm. An arrow indicates the shift of the minimum with RI.

In Figure 4, in turn, the spectral response of a reference µIMZI is shown. This structure was micromachined separately for comparison. The microcavity has the same diameter *d* = 60 µm as the µIMZIs fabricated later in the LPGs. In general, we observe here a shift of the transmission minima towards shorter wavelengths with increasing RI values in the cavity. The substantiation of the spectrum evolution was presented in our previous work [27]. It is worth mentioning that due to the micromachined interferometric structure, the overall transmission dropped by ca. 7–10 dB. This effect is induced by the formation of the µIMZI cavity, which makes direct interaction between the fiber core and the external medium possible. The RI sensitivity of the presented µIMZI reaches 15,000 nm/RIU.

### 3.2. RI Sensing with LPG-µMZI Structures

**Figure 5:**
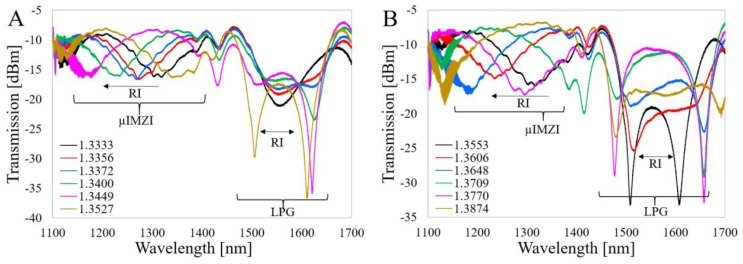
Spectral response of the LPG-µIMZI structure to RI in ranges: (**A**) from 1.3333 to 1.3527 RIU and (**B**) from 1.3553 to 1.3874 RIU. The effects corresponding to the LPG and µIMZI are marked in the figures.

Figure 5A,B shows the spectral response to RI in its different ranges for the LPG after µIMZI micromachining, i.e., with the microcavity in the middle of the LPG.

**Figure 6:**
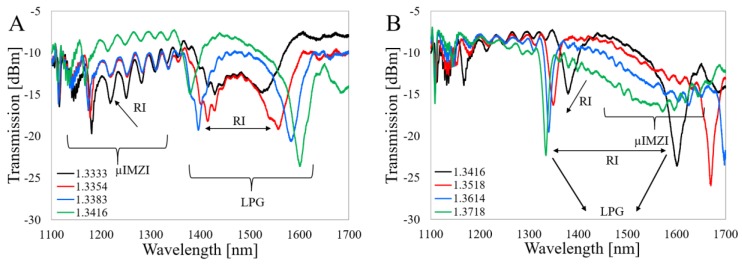
Spectral responses of the Al_2_O_3_-LPG-µIMZI structure to RI in its range (**A**) from 1.3333 to 1.3416 RIU and (**B**) from 1.3416 to 1.3718 RIU. The effects corresponding to the Al_2_O_3_-LPG and µIMZI are marked.

It is interesting that the LPG still ordinarily responds to the RI despite a significant discontinuity in the fiber cladding. Given that the LPG’s working principle relies on the cladding modes, it is perhaps counter-intuitive that the microcavity had no direct effect on the LPG response. The micromachining process led to an increase in the overall insertion loss (transmission dropped by ca. 10–14 dB). The presence of the cavity also manifests itself in the oscillatory character of the spectrum, which in this case is caused by scattered waves at the cavity interferences. Finally, two specific ranges in the spectrum are evident, above and below 1400 nm. The higher part resembles the spectrum of the LPG before micromachining (Figure 3A), while the lower one is dominated by the response characteristic to the µIMZI (Figure 4). Based on the curves shown in Figure 5 and Figure 6, we can state that the LPG-µIMZI response is a composite of the LPG and µIMZI spectra. Figure 5 shows that with an increase of the RI, the spectral response of the combined device is similar to the responses of the LPG and µIMZI working independently, which is more evident for smaller values of RI. Specifically, we observe an increase of the resonance wavelength separation in the range of 1400–1700 nm, which corresponds to the profile of the LPG. There is also a blue shift of the minima in the 1200–1400 nm range which, in turn, typically corresponds to responses of the µIMZI (Figure 5A). For higher values of RI, the second minima, characteristic for the µIMZI, appear at the higher wavelengths and mingle with the response induced by the LPG. This causes distortions in the spectrum and makes the two effects interfere with one another (Figure 5B). The dominant regions for each effect are still distinguishable, but very much disturbed, especially in the case of the minima characteristic for the LPGs. The interfering effects in some RI ranges are critical and this fact needs to be taken into consideration while designing the device.

Next, the RI-induced spectral evolution for the Al_2_O_3_-LPG with microcavity was investigated (Figure 6).

As in the case of the LPG, the overall transmitted power after micromachining is significantly reduced. The transmission dropped by ca. 7–11 dB. Thanks to the higher sensitivity of the Al_2_O_3_-LPG, the effect is even more noticeable at higher RI values. On the other hand, the more sensitive the LPG structure is, the more distortion and undulations appear in the spectrum after the micromachining. Although it was always possible to determine one of the LPG’s minima, as well as the minimum for the µIMZI, the obtained spectrum was not that smooth and well pronounced as in the case of the sensors working separately.

A few possible causes for the origins of these distortions are, e.g., exciting additional modes, or introducing a phase shift to the already existing one. It is also worth reminding that both the sensing structures were made in a standard single-mode fiber where the cutoff wavelength is λ = 1260 nm. Below this value, the fiber works in a multimode regime and the high-frequency oscillations in the spectrum are observed mainly in the short wavelength region. As shown in Figure 6A, the minimum at around 1200 nm corresponding to the µIMZI diminishes with RI, i.e., moves towards the shorter wavelengths and cannot be further observed due to the cut-off wavelength of the fiber. Nevertheless, the second minimum can be seen above RI of 1.3416 RIU at about 1600–1700 nm (Figure 6B).

It can be concluded that the results obtained for the two types of LPGs with µIMZIs differ only quantitatively. The similarities are explained by the fact that both have the same effect and the same general principle of operation, while the differences have several reasons. The spectra shown in Figure 6 indicate that the minimum corresponding to the µIMZI is in the short wavelength region. The second minimum, in contrast to the LPG, emerges for relatively lower RI values. Moreover, due to the considerably higher sensitivity of the Al_2_O_3_-LPG, its resonances shift significantly more with the RI and the µIMZI effect is more pronounced and less distorted than for higher RI in case of the LPG. It might be expected that a RI range exists for which the distortion makes discrimination of the effects difficult. However, in the investigated cases during the whole experiment, i.e., for the entire RI range, it was always possible to determine one of the LPG’s minima, as well as the minimum of the µIMZI. It is important to note that in this case, i.e., Al_2_O_3_-LPG, the exact definition of the location of the minima requires additional signal processing, e.g., curve fitting or pattern recognition. Furthermore, along with some additional signal processing, the full-width half minimum (FWHM) parameter could also be improved, especially for the µIMZI part of the spectrum. Even though its reduction requires additional fine-tuning of the sensor, such as reactive ion etching as reported in our previous work [28]. In this discussion, we must acknowledge that also the placement of the cavity might influence the transmission spectrum of the device. In the case of both structures, despite the significant discontinuity in the fiber cladding, which sustains the cladding modes, the LPG-related effect remains valid and the cavity does not affect the output of the sensor significantly.

From the above discussion, we can conclude that the two effects, the first stemming from the LPG and the second from the µIMZI, seem to be independent. Furthermore, we have seen that in lower RI ranges they do not affect each other and can be well separated. This separation of effects may be used to reduce the cross-sensitivity of the proposed device. Since the lower RI range of operation is essential in many applications, including bio-sensing, the combined structure could well serve for taking measurements where two different RI sensitivity effects are expected. Experiments such as those reported here have been carried out previously for both platforms separately [14,19], but never for a combination of the platforms. The prospect of being able to simultaneously detect and distinguish the earlier mentioned different sensitivities (surface and volume) effects using a two-in-one platform serves as a continuous incentive for ongoing studies and motivates future research.

### 3.3. Monitoring of RI at the Surface with the LPG-µMZI

**Figure 7:**
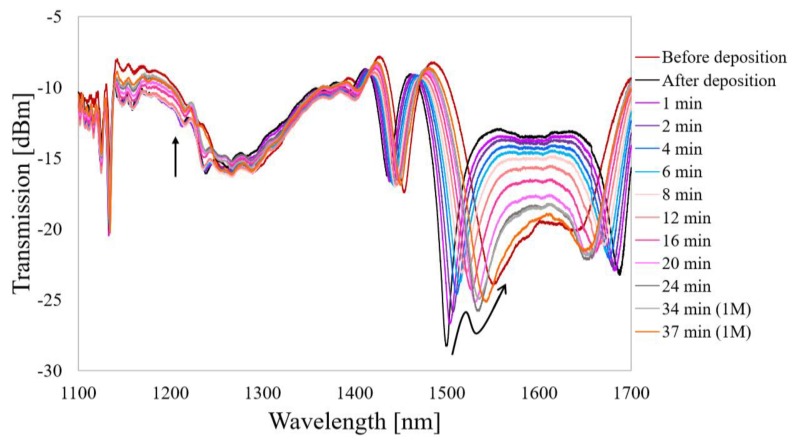
The response of LPG-µIMZI to Al_2_O_3_ film etching recorded for measurements in water. The plot shows the evolution of the transmission spectrum during the process of the response before the deposition. Due to the low effectiveness of the etching process, the last two etching rounds were performed in 1 M NaOH.

In the previous sections, we discussed the high *S_V_* of the sensor. However, the primary motive behind the combination of the LPG and µIMZI was rather a determination of the difference between *S_V_* and *S_S_*. Based on the working principles of each platform it can be stated that the LPG is best suited for detecting surface RI changes, while the µIMZI excels in volume RI measurements, where the surface effect can be ignored to some extent. To prove our hypothesis and simulate the biological film formation, we deposited a high-RI thin overlay of Al_2_O_3_ using the ALD method on the entire LPG-µIMZI. ALD provided a highly controllable and uniform 30 nm layer all over the LPG-µIMZI sensor. The Al_2_O_3_ can be dissolved in both highly concentrated acids and alkalis, which enables studying the response of the sensor to different thicknesses of the thin film [26]. In Figure 7 the influence of the Al_2_O_3_ etching process on the spectral response of the LPG-µIMZI is shown. The arrows indicate the change of the spectral response with the progression of the etching process.

The most apparent change concerns the LPG part of the spectrum. LPGs are highly sensitive towards external RI including even the thinnest overlay formed on their surface. Any change of the thickness of the nanocoating manifests itself as a shift of the resonance wavelength, and it was observed after the deposition process. Both resonances shifted away from each other for about 50 nm from the initial stage. Moreover, the LPG operates in the proximity of the DTP. Thus, the deposition process splits both resonances and makes them apparent and defined. Slow etching in 10 mM NaOH induced the shift of the spectrum (indicated by black arrows) and with time the resonances were getting close to each other, and finally, they got back to the initial working point. In contrast to the response from the LPG, the µIMZI part of the spectrum only slightly reacted by the deposited Al_2_O_3_ overlay. We can observe the changes mainly in terms of the transmission amplitude. The properties of the deposited layer—its RI and thickness—did not change the working conditions of the µIMZI. In conclusion, if we are targeting detection of surface changes (ca. tens of nm overlays), it will be too small to affect the volume sensitivity of the µIMZI part of the sensor and in effect, it will stay unrecognizable to the µIMZI, or it will induce changes just in terms of transmission amplitude. However, combined with surface-sensitive LPG, the sensor can stand as a perfect tool to measure the growth of an overlay and, for example, simultaneous fluctuations of the external RI. This, in turn, could find the application in the detection of biological targets such as DNA aptamers, which are a key target in medical diagnostic tests.

**Figure 8:**
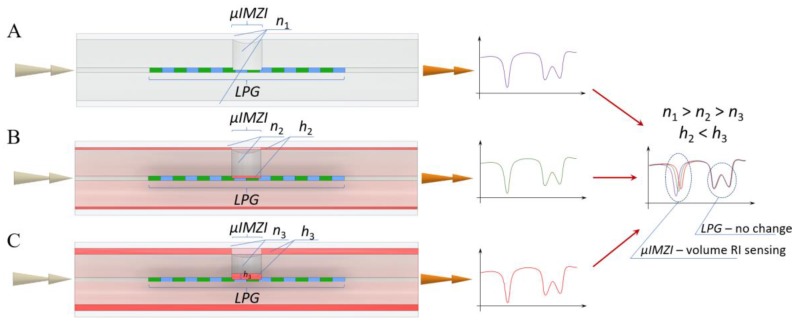
A schematic showing LPG-µIMZI influenced by (**A**) high external RI, (**B**) thin film and lower external RI, and (**C**) thick film and the lowest external RI. For this specific combination of external RIs (n_1_, n_2_, and n_3_) and film thicknesses, only the response of µIMZI would allow discriminating the cases.

Figure 8 presents a schematic comparison of three cases when the use of combined LPG-µIMZI would allow to deliver enriched information about the analyzed thin film, including biofilm, and the surrounding liquid. Figure 8A shows a situation when both the LPG and µIMZI are influenced by a high-RI liquid (n_1_). Figure 8B, in turn, presents a case where a very thin layer (h_2_), such as a biological film is deposited, and the external RI is lower than in the case shown in Figure 8A (n_2_ < n_1_). The last example (Figure 8C) concerns a thicker layer (h_3_ > h_2_) and the lowest RI (n_3_ < n_2_). For the LPG, the measurement will be a superposition of effect coming from RI, the thickness of the nanolayer, and to some extent RI of the surrounding medium. Thus, on the LPG part of the spectrum for specific values of n_1_, n_2_, and n_3_, as well as h_2_ and h_3_ there will be no or slight difference between thin and thicker film surrounded by higher and lower external RI. Only the contribution from µIMZI would allow to discriminate the cases and identify changes in RI and, in consequence, changes in film growth, too. During the biological experiments, the discrimination of such differences is crucial, e.g., the thickness of the film may correspond to the concentration of biological targets, while external RI indicates proper removal of excess of the unbound targets. Thus, the proposed sensing combination enables clear identification of the changes at the surface and in its proximity, as well as further interpretation of the results.

### 3.4. Temperature Sensitivity of LPG-µIMZI

**Figure 9:**
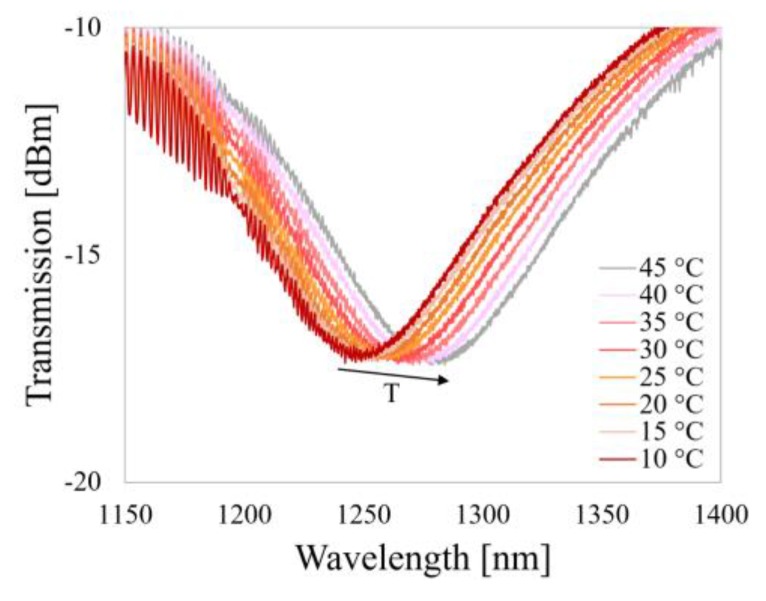
Transmission spectra of µIMZI at different temperatures (T) of water in the cavity.

In the preceding Section 3.2 and Section 3.3, it was shown that the combined structures work independently and do not affect each other’s operation in any significant way. This can be even more interesting when one realizes that in addition to a slight reaction to the thin overlay deposition, in contrast to the single LPG, the µIMZI is almost T-insensitive [18]. To demonstrate the T-insensitive RI sensing with the µIMZI, the reference µIMZI was placed in a T-controlled cell and immersed in water. The transmission spectrum was monitored while the T was gradually increased from 10 to 45 °C with a 5 °C step. The spectra obtained during these measurements show that with an increase of T, the minimum shifted towards longer wavelengths (Figure 9).

The RI of the fiber core, as well as diameter of the microcavity, are treated as constants over the range of the T applied during the described experiment (10–45 °C) considering the thermo-optic coefficient (6.3 × 10^−6^/°C) and thermal expansion coefficient (0.55 × 10^−6^/°C) of the fiber materials [29,30]. The obtained T sensitivity of the µIMZI in water is 1.2 nm/°C at ca. 1250 nm and is induced mainly by RI sensitivity of the structure which is ≈15,000 nm/RIU. Since the thermo-optic coefficient of water reaches −1 × 10^−4^ RIU/°C and is two orders of magnitude higher than that of fused silica (6.3 × 10^−6^/°C), we can conclude that the observed shift of the minimum is induced almost exclusively by the change in the RI of water caused by T variation.

**Figure 10:**
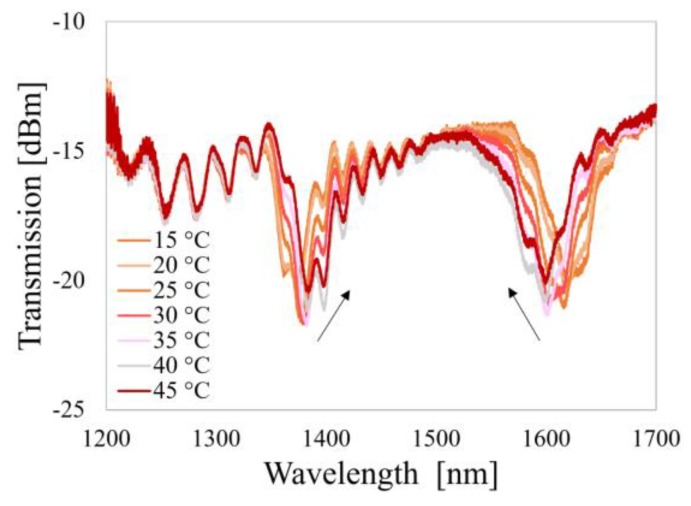
Spectral responses of the Al_2_O_3_-LPG-µIMZI structure in water to variations of T.

In Figure 10 evolution of Al_2_O_3-_-LPG-µIMZI transmission spectra with T is presented. Here, we see that with the increase of T, the spectral distance between the resonances of the LPG decreases, while the part dominated by the microcavity response barely changes. Assuming that the two effects are independent and that the change of the spectrum caused by the µIMZI is relatively small when compared to that caused by the LPG, we can infer that the sensor is suited for highly accurate RI measurements in cases where the T of the investigated medium varies.

Multiparameter sensing is often unavoidable to acquire desired information on the performance of a chosen system. Thus, in recent years many configurations have been presented, especially for T and RI sensing. However, just two other sensors are incorporating a grating and in-fiber cavities. The first sensor was created by combining the micromachined cavities in-between two FBGs [31]. The second was made by a combination of FBG with the µIMZI [6]. In both cases, the FBGs provided very high sensitivity. However, because of the combination, as indicated in the introduction, differences in the values of the traced wavelength shifts limit the practical application of the sensors. The design and manufacturing method of the first-mentioned sensor also highly bounds its performance. Two microcavities made in one fiber significantly weakened the sensing structure. Besides, the reproducibility of the sensor is questionable due to the Excimer laser processing combined with highly uncontrollable hydrofluoric acid etching. Regardless of the combination, both sensors provided the information about T and RI. Therefore, they cannot be compared in terms of surface and volume sensitivities. This is the first sensor to date which has been considered for such application.

## 4. Conclusions

This work presents a device containing LPG and µIMZI in the same single-mode optical fiber. Two LPGs, tuned towards high RI sensitivity by fiber cladding etching (2000 nm/RIU) and Al_2_O_3_ nanocoating deposition (9000 nm/RIU), were later modified by fabrication of cylindrically shaped cavities. These microcavities with diameters of *d* = 60 µm were micromachined in the middle of each of the 5-cm long LPGs. During the experiments, it was found that in both cases, they responded as two independently working sensors, one associated with the grating and the other with the microcavity. At some values of the RI, an overlapping took place. The problem of distortions or overlapping can be overcome by additional signal processing, e.g., curve fitting or pattern recognition, however, it needs further studies as it may limit the operation range of the sensor. Nevertheless, this effect has no significant impact on the independent operation of the sensors in the specified lower RI ranges, which are required by many applications, e.g., bio-sensing. Although neither of the platforms improved the other one, they perfectly complemented each other and they both had exceptional RI sensitivity (LPG at surface and μIMZI in volume). Thus, this combination allowed to obtain more in-depth information about the measurand through multi-parameter sensing. Especially small bio-targets, such as DNA aptamers, should be considered where the LPG part would be responsible for specific label-free recognition and the µIMZI’s signal would be responsible for tracing the RI and its fluctuations caused by, e.g., temperature distortions.
